# Diseases of the Ear, Nose and Throat, and Their Accessory Cavities

**Published:** 1899-05

**Authors:** 


DISEASES OF THE EAR, NOSE AND THROAT, AND THEIR ACCESSORY CAVITIES By SETH SCOTT BISHOP, M. D., D. C. L., LL. D., Professor of Diseases of the Nose, Throat and Ear in the Illinois Medical College; Professor in the Chicago Post-Graduate Medical School and Hospital; Surgeon to the Post-Graduate Hospital, etc. Second edition, thoroughly revised and enlarged. Illustrated. Philadelphia: The F. A. Davis Company. 1898.

This is a concise, well written manual, admirably suited to the needs of the student. The subjects are systematically and clearly presented and the work embraces the essentials of otology, rhinology and laryngology as understood at the present time. The illustrations are numerous, many of them being colored, and serve to make the text more easily understood. To the student who desires a compact and trustworthy text-book on diseases of the ear, nose and throat in one volume, this work is to be commended. A. A. H.

